# Fibroblast surface-associated FGF-2 promotes contact-dependent colorectal cancer cell migration and invasion through FGFR-SRC signaling and integrin α_v_β_5_-mediated adhesion

**DOI:** 10.18632/oncotarget.3883

**Published:** 2015-05-05

**Authors:** Sarah Knuchel, Pascale Anderle, Patricia Werfelli, Eva Diamantis, Curzio Rüegg

**Affiliations:** ^1^ Department of Medicine, Faculty of Science, University of Fribourg, Fribourg, CH-1700, Switzerland; ^2^ Swiss Institute of Bioinformatics, Lausanne, CH-1000, Switzerland; ^3^ Department of Oncology, University Medical Center (CHUV), University of Lausanne (UNIL), Lausanne, CH-1011, Switzerland; ^4^ Swiss National Centre of Competence in Research Molecular Oncology, Swiss Institute for Experimental Cancer Research, Ecole Polytechnique Fédérale de Lausanne (EPFL), School of Life Sciences, Lausanne, CH-1015, Switzerland; ^5^ Division of Clinical Pathology, Institute of Pathology, University of Bern, Bern, CH-3010, Switzerland

**Keywords:** colorectal cancer, fibroblast, FGF-2, integrin, SRC

## Abstract

Carcinoma-associated fibroblasts were reported to promote colorectal cancer (CRC) invasion by secreting motility factors and extracellular matrix processing enzymes. Less is known whether fibroblasts may induce CRC cancer cell motility by contact-dependent mechanisms. To address this question we characterized the interaction between fibroblasts and SW620 and HT29 colorectal cancer cells in 2D and 3D co-culture models *in vitro*. Here we show that fibroblasts induce contact-dependent cancer cell elongation, motility and invasiveness independently of deposited matrix or secreted factors. These effects depend on fibroblast cell surface-associated fibroblast growth factor (FGF) -2. Inhibition of FGF-2 or FGF receptors (FGFRs) signaling abolishes these effects. FGFRs activate SRC in cancer cells and inhibition or silencing of SRC in cancer cells, but not in fibroblasts, prevents fibroblasts-mediated effects. Using an RGD-based integrin antagonist and function-blocking antibodies we demonstrate that cancer cell adhesion to fibroblasts requires integrin α_v_β_5_. Taken together, these results demonstrate that fibroblasts induce cell-contact-dependent colorectal cancer cell migration and invasion under 2D and 3D conditions *in vitro* through fibroblast cell surface-associated FGF-2, FGF receptor-mediated SRC activation and α_v_β_5_ integrin-dependent cancer cell adhesion to fibroblasts. The FGF-2-FGFRs-SRC-α_v_β_5_ integrin loop might be explored as candidate therapeutic target to block colorectal cancer invasion.

## INTRODUCTION

Colorectal cancer (CRC) is the third most common cancer in the world. Despite advances in treatments, existing therapies are of limited effectiveness once cancer has become invasive or metastatic [1]. Understanding the molecular mechanisms underlying CRC cell invasion may lead to the identification of patients at high risk for disease progression and to the discovery of novel therapeutic targets and strategies. During tumor progression cancer cells modify the surrounding normal tissue to create a microenvironment supporting tumor growth and progression [2]. The tumor microenvironment consists of a variety of tumor-recruited or locally activated cells, such as bone marrow-derived cells, immune cells and carcinoma-associated fibroblasts (CAF) [3]. Dynamic reciprocal interactions between tumor cells and their microenvironment contribute to tumor cell survival, proliferation, invasion and metastasis and are therefore increasingly considered as potential targets for novel therapeutic strategies [4].

Despite the fact that CAF are among the most abundant and crucial cells of the tumor microenvironment, these cells remains poorly defined due to their inherent plasticity, heterogeneity and different origins, as well as the lack of single, universal specific markers and their pleiotropic functions [5–8]. Nevertheless, several distinct morphological properties and functions of CAF have been described and characterized. CAF are contractile cells commonly characterized by the expression of multiple activation markers, including α-smooth muscle actin (SMA), vimentin (VIM) or fibroblast-activated protein (FAP) [9, 10]. The presence of CAF is associated with aggressive progression and poor prognosis in several cancer types [11–13]. Experimental and clinical evidences indicate that CAF regulate a number of tumor-promoting functions, including angiogenesis, invasion and metastasis [9, 14]. The higher CAF density is generally found at the invasive front of the tumor [10], and several studies have shown that CAF promote CRC progression though multiple mechanisms [10, 11, 15]. Firstly, CAF produce soluble factors, such as hepatocyte growth factor/scatter factor (HGF) and platelet-derived growth factor (PDGF), promoting survival, proliferation and motility of cancer cells and stimulating the recruitment and activation of other cells in the tumor microenvironment, in particular inflammatory cells [16, 17]. Secondly, CAF induce angiogenesis and enhance vascular permeability through the secretion of vascular endothelial growth factor (VEGF) [18]. Thirdly, CAF promote cancer cells invasion by modifying extracellular matrix (ECM) through the deposition of matrix proteins, including tenascins [11, 19], and the secretion of matrix metallo-proteases (MMPs) resulting in altered tensile forces, gradients and release of bioactive fragments promoting cancer cell motility [11, 20]. In spite of the fact that CAF can be in contact with cancer cells during stroma invasion [19] only little is known on the putative role of direct CAF-cancer cell contact in promoting cancer cell motility and invasion.

Here we have addressed this question by characterizing the interaction between fibroblasts and CRC-derived cell lines SW620 and HT29 *in vitro* using 2D and 3D co-culture models. Presented results demonstrate that fibroblasts promote CRC cell migration and invasion through direct cell-cell contact involving fibroblast cell surface associated FGF-2 and FGF receptors (FGFR) - integrin α_v_β_5_-SRC dependent signaling in cancer cells.

## RESULTS

### Fibroblasts promote SW620 and HT29 CRC cell elongation and motility

**Figure 1:**
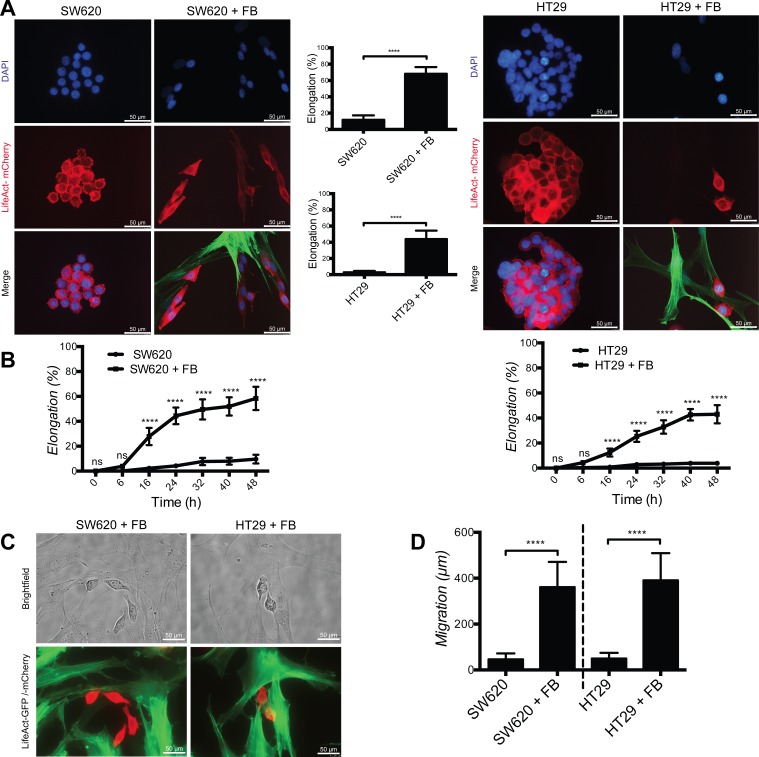
Fibroblasts induce cancer cell elongation and motility **A.** Representative images of SW620 and HT29 cancer cells (red) in absence or presence of dermal fibroblasts (+FB, green). Bar graphs represent quantification of cancer cells elongation. **B.** Time course of cancer cells elongation quantification. **C.** Representative live images of adhesion between fibroblasts (green) and cancer cells (red). **D.** Quantification of cancer cells motility during 48 hours in presence or absence of fibroblasts. All data are represented as mean +/− SD.

To monitor the effect of fibroblasts on CRC cell we cultured the CRC-derived cell lines SW620 and HT29 in the absence or presence of skin-derived fibroblasts. When cultured alone, SW620 and HT29 have a rather rounded morphology, while after 48 hours culture in the presence of fibroblasts they acquire an elongated morphology (Fig. 1A). Time lapse imaging revealed that only cancer cells establishing contacts with fibroblasts develop pseudopodia at the attachment site and progressively acquire an elongated morphology over time (about 70% of SW620 and 50% of HT29 compared to less than 10% in the absence of fibroblasts) (Fig. 1B and 1C). Concomitant to elongation, cancer cells cultured with fibroblasts massively increased their motility, as monitored by tracking the distance travelled by individual cells (Fig. 1D).

These results demonstrate that fibroblasts induce colon cancer cell elongation and motility.

### Cultured dermal, normal colon or colon cancer fibroblasts have equivalent gene expression and activation profiles and induce comparable cancer cell elongation and motility

**Figure 2:**
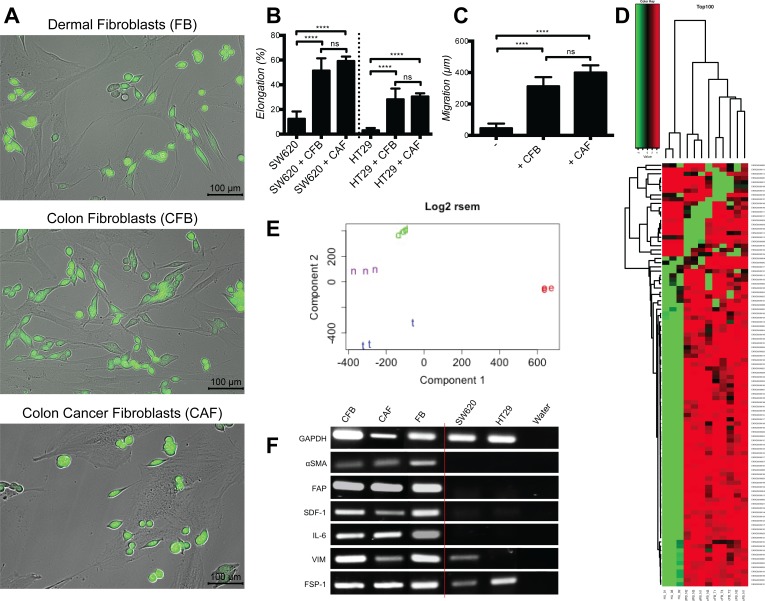
Cultured dermal, colon and colon cancer associated fibroblasts induce similarly cancer cell elongation and motility and have equivalent gene expression and activation profiles **A.** Representative images of SW620-GFP co-cultured with dermal fibroblasts (FB), normal colon fibroblasts (CFB) and colon cancer associated fibroblasts (CAF). **B.** Quantification of cancer cell elongation with CFB and CAF, represented as mean +/− SD. **C.** Quantification of SW620 motility during 48 hours culture with CFB and CAF, represented as mean +/− SD. **D.** Self-organizing heat-maps of the top 100 genes with greatest variability across all samples, showing similar expression profile for Colon Normal Fibroblasts (cFB_N), Colon Cancer Fibroblasts (cFB_T) and Dermal Fibroblasts (dFB_N), but highly different profiles compare to HUVEC (HU). **E.** PCA plot demonstrating that Colon Normal Fibroblasts (n), Colon Cancer Fibroblasts (t) and Dermal Fibroblasts (d) are similar, while they greatly diverge from HUVEC (e). **F.** PCR expression analysis of fibroblast activation markers.

Next we tested whether fibroblasts isolated from normal colon (CFB) or colon cancer (CAF) tissues were also able to induce cancer cell elongation and motility. Indeed, CFB and CAF induced SW620 and HT29 elongation and motility to extents comparable to those exerted by dermal fibroblasts (Fig. 2A-2C). The fact that dermal fibroblasts and CFB were able to induce these effects on CRC cells was unexpected, as previous studies demonstrated that only freshly isolated CAF, but not normal fibroblasts, induced cancer progression *in vivo* [21, 22].

To explain these similar properties, we hypothesized that fibroblasts cultured and expanded *in vitro* might acquire common functional capabilities regardless of their *in vivo* origin. To substantiate this hypothesis we performed gene expression profiling analyses on CFB, CAF and dermal fibroblasts (FB). Self-organizing heat-maps of the top 100 differentially expressed genes revealed that all fibroblasts display a very similar expression profile (Fig. 2D). As comparison, umbilical cord endothelial cells (HUVEC) have a clearly different gene expression profile. Moreover Principal Component Analysis (PCA) confirmed that all tree fibroblasts populations cluster together and clearly segregate from HUVEC (Fig. 2E). In addition volcano plot analysis confirms the results (data not shown).

To further strengthen these observations we monitored transcripts profiles for fibroblasts activation markers typically observed in CAF [10, 15]: α-SMA, FAP, stroma-derived factor (SDF)-1, interleukin-6 (IL-6), VIM and fibroblasts specific protein (FSP)-1. Transcripts for all these markers were similarly expressed across all fibroblasts populations, thereby indicating equivalent activation states (Fig. 2F). FSP-1 and VIM were also expressed in cancer cells, consistent with previous reports [23, 24].

**Figure 3:**
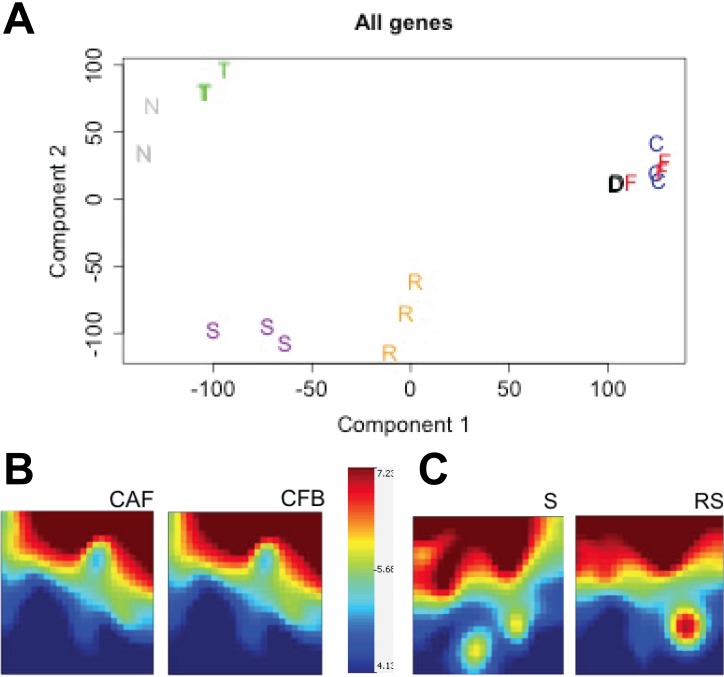
Normal and colon cancer stroma have different gene expression profiles *in vivo* while fibroblasts isolated thereof and cultured *in vitro* have similar profiles **A.** PCA plot representing similar expression profile for *in vitro* cultured colon normal fibroblasts (F), colon cancer fibroblasts (C) and the lung fibroblasts MRC5 cell line (D). In contrast, profiles of laser-capture micro-dissection normal stroma (S) and reactive stroma (R) segregate distinctly along component 1. Tumor cells (T) and normal colon epithelial cells (N) have completely different profiles compared to stromal cells. **B.** Self-organizing maps of cultured CAF and CFB showing very similar genomics profiles. Color scale shows A average. **C.** Self-organizing maps showing different genomics profiles of reactive stroma (RS) and normal stroma (S) freshly dissected by laser-capture micro-dissection.

To collect further evidence supporting the notion that *ex vivo* culture alters gene expression profile in fibroblasts, we performed gene expression profiling analyses on CAF and CFB and compared them to expression profiles of laser-capture micro-dissected CRC stroma and normal colon stroma. PCA demonstrate that laser micro-dissected normal stroma and reactive stroma have different expression profile, while cultured CAF and CAB have similar expression profiles (Fig. 3A). Normal colonic epithelial cells and cancer segregate separately. Self-organizing maps of genomics profiles further confirmed that expression profiles of cultured CAF and CFB were indistinguishable (Fig. 3B), while genomics profiles of laser-capture micro-dissected tumor stroma and normal stroma showed clear differences (Fig. 3C) consistent with the notion that *in vivo* differences in gene expression are blunted by *in vitro* cell culture.

Taken together these results indicate that cultured dermal fibroblasts, CFB and CAF are in a similar state of activation and are equally effective in inducing colon cancer cells elongation and motility in our model. Since CFB and CAF have reduced proliferative capacity *in vitro* compared to dermal fibroblasts (data not shown), we performed most of the experiments using dermal fibroblasts whereas CFB and CAF were used in selected validation experiments.

### Cancer cell elongation and migration require direct contact with living fibroblasts

**Figure 4:**
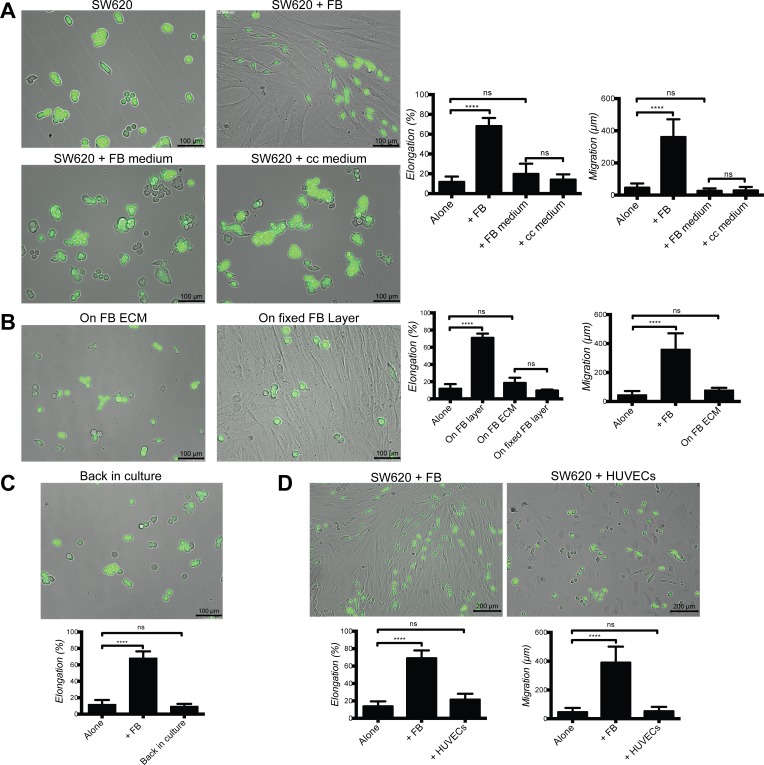
Fibroblast-induced elongation and motility of cancer cells require direct contact with living fibroblasts **A.** Representative images of SW620-GFP cultured in the presence or absence of fibroblasts, fibroblasts and co-culture (cc) conditioned media. Bar graphs represent quantification of SW620 elongation and motility for 48 h. **B.** Representative images of SW620-GFP cultured on ECM deposited by fibroblasts and on a fixed fibroblast layer. Bar graphs represent quantification of SW620 cell elongation and motility for 48 h. **C.** Representative image and quantification of elongation of SW620-GFP returned to culture after separation from established co-culture. **D.** Representative images and quantification of elongation and motility of SW620-GFP cultured with HUVECs and fibroblasts for 48 h. All data are represented as mean +/− SD.

As elongated cancer cells were closely associated with fibroblasts we asked the question whether direct contact was required or whether soluble factors or extracellular matrix were sufficient in mediating such effects. To this purpose SW620 and HT29 were exposed to conditioned medium from fibroblast cultures or fibroblast-cancer cell co-cultures. Both conditions were ineffective in inducing cancer cell elongation or motility (Fig. 4A and Supplementary Fig. 1A). In addition, cancer cells cultured on an ECM deposited by fibroblasts, as demonstrated by the detection of fibronectin (Supplementary Fig. 2A), or on fixed fibroblasts, did not acquire elongation or motility (Fig. 4B and Supplementary Fig. 1B). Elongated cancer cells isolated from an established co-culture and re-plated in the absence of fibroblasts reverted to a round morphology (Fig. 4C and Supplementary Fig. 1C). The purity of cancer cell fraction isolated from co-culture with fibroblasts was over 95% (Supplementary Fig. 2B and 2C). Importantly cancer cell co-cultured with HUVECs did not result in any effect on elongation or migration (Fig. 4D and Supplementary Fig. 1D). Exposure of SW620 or HT29 to exogenous FGF-2, EGF and TGFβ, three cytokines known to induce cancer cell motility, failed to induce elongation and migration (Supplementary Fig. 3A and 3B).

From these experiments we concluded that fibroblast-induced SW620 and HT29 cell elongation and motility require continuous contact with living fibroblasts.

### Fibroblasts promote contact-dependent cancer cell invasion

**Figure 5:**
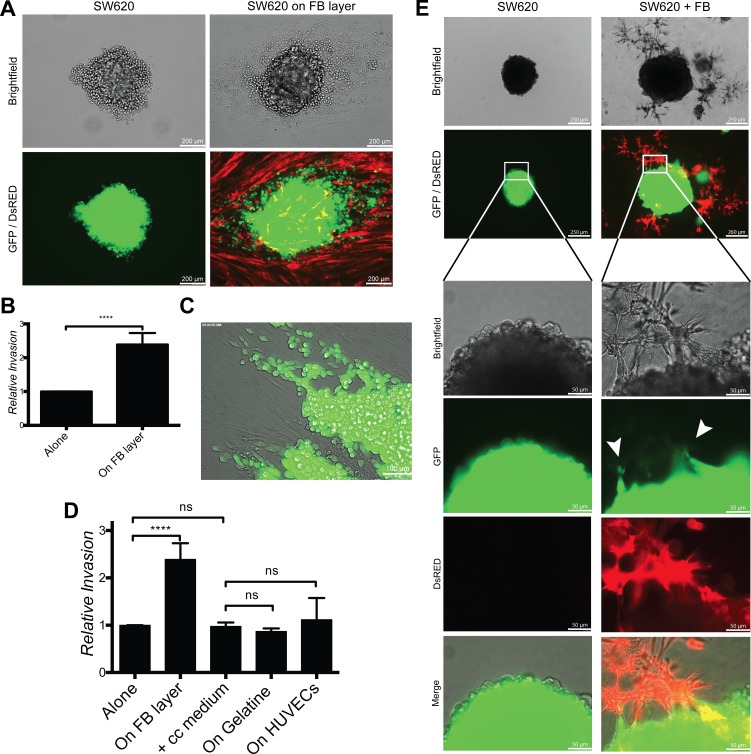
Fibroblasts induce cancer cell invasion in 2D and 3D conditions **A.** Representative images of SW620-GFP invasion from spheroids cultured for 4 days alone or on a fibroblast-DsRED layer (2D condition). **B.** Quantification of SW620 invasion of experiment in panel A, represented as mean +/− SD. **C.** Image of SW620-GFP invading the fibroblast layer out of a spheroid. **D.** Quantification of SW620-GFP spheroid invasion after 4 days under indicated conditions represented as mean +/− SD. **E.** Representative images of SW620-GFP 3D spheroids in presence or absence of fibroblasts-DsRED after 4 days. Arrows are indicating invasion areas.

To test whether fibroblasts can also promote cancer cell invasion, we used 2D and 3D spheroid assays. For the 2D assay, SW620 spheroids were cultured on a fibroblasts monolayer for 4 days. Fibroblasts induced SW620 cell scattering and migration out of the spheroids (Fig. 5A-5B). SW620 migrating along fibroblasts acquired an elongated morphology (Fig. 5C). Culturing tumor spheroids alone, with co-culture conditioned medium, on a gelatin substrate or on a HUVEC monolayer did not induce cancer cell invasion (Fig. 5D and Supplementary Fig. 3C), thereby confirming that the effect was fibroblast specific and contact dependent. We then tested for fibroblasts-induced cancer cell invasion in 3D conditions by embedding SW620 cells spheroids in fibroblasts-supplemented Matrigel. Fibroblasts migrated toward the spheroids, established contacts with cancer cells and induced SW620 invasion into the surrounding matrix. SW620 spheroids embedded in Matrigel without fibroblasts did not develop invasive properties (Fig. 5E).

From these experiments we concluded that fibroblasts induce contact-dependent cancer cell invasion.

### Cancer cell elongation, migration and invasion require FGFR signaling

**Figure 6:**
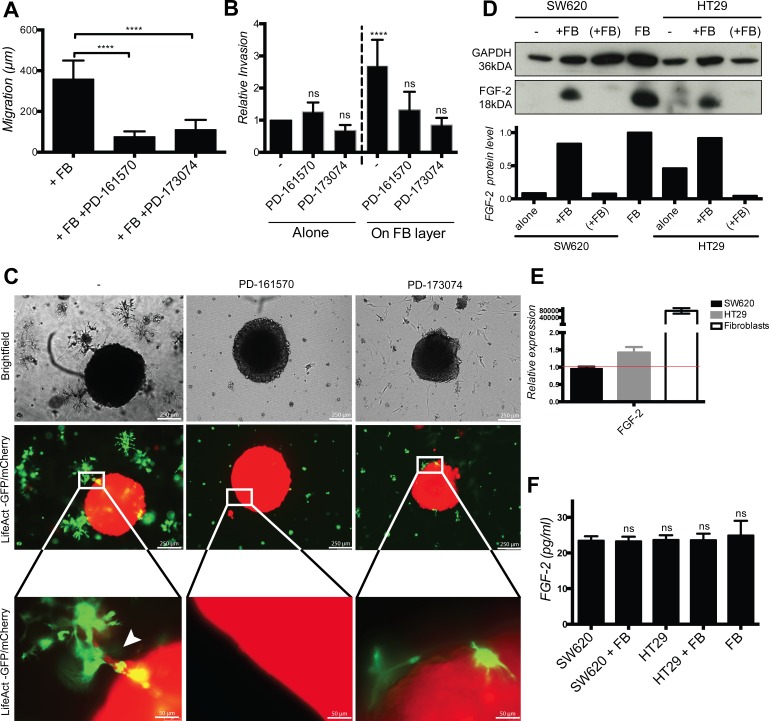
Cancer cell migration and invasion depends of fibroblast-cell surface associated FGF-2 and FGFR signaling **A.** Quantification of SW620 motility cultured with fibroblasts in the absence or presence of PD-161570 and PD-173074 FGFR inhibitors for 48 h. **B.** Quantification of SW620 2D spheroid invasion cultured as indicated after 4 days. **C.** Representative images of SW620-LifeAct-mCherry 3D spheroid invasion cultured with fibroblasts-LifeAct-GFP in the absence or presence of FGFR inhibitors after 4 days. **D.** Western blotting analysis of FGF-2 protein in fibroblasts, cancer cells alone and in co-culture with (“(+FB)”) or without (“+FB”) cancer cell separation. **E.** Relative expression of FGF-2 mRNA in fibroblasts, SW620 and HT29 determined by RT-PCR. **F.** ELISA quantification of soluble FGF-2 in cell culture supernatants as indicated. All data are represented as mean +/− SD.

In order to define the molecular mechanism involved in contact-dependent fibroblast-induced cancer cell elongation, migration and invasion, we applied pharmacological inhibitors of several known invasion-promoting kinases (i.e. PI3K/AKT, c-MET, MAPK/ERK, EGFR or FGFR) to co-cultures. Of all the tested inhibitors, only the FGFR inhibitors PD-161570 and PD-173074 prevented fibroblasts-induced cancer cell elongation (Supplementary Fig. 4A and 4B). PD-161570 and PD-173074 also inhibited cancer cell migration (Fig. 6A and Supplementary Fig. 4C) and invasion in the 2D (Fig. 6B) and 3D (Fig. 6C) assays.

Exogenous soluble FGF-2 did not induce cancer cell elongation (Supplementary Fig. 3A and 3B) and FGF-2 protein was readily detected by Western blotting in fibroblast but not in cancer cell lysates (Fig. 6D). mRNA level of FGF-2 was also much higher in fibroblasts compared to cancer cells (Fig. 6E), however FGF-2 protein levels were identical in culture supernatants of fibroblasts, cancer cells and co-cultures (Fig. 6F). Co-culture with cancer cells further enhanced fibroblasts FGF-2 mRNA expression by about 3 fold (Supplementary Fig. 4D).

### Cancer cells adhesion on fibroblast requires fibroblast surface-associated FGF-2, FGFR signaling and α_V_β_5_ integrin ligation

**Figure 7:**
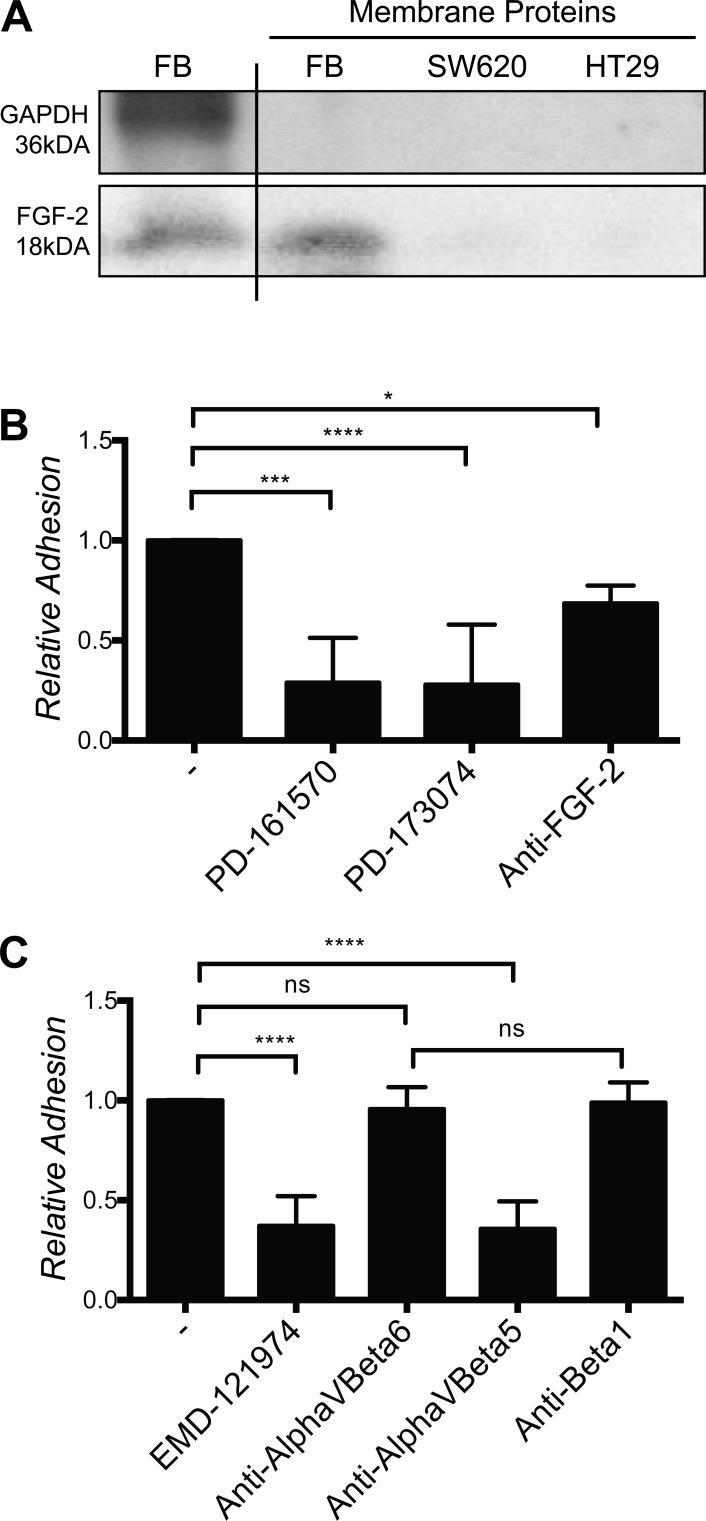
Fibroblasts cell surface-associated FGF-2, FGFR and αvβ5 integrin are required for SW620 cell adhesion to fibroblasts **A.** Western blot of FGF-2 from normal cell lysate and from the extracted membrane proteins fraction. **B.** Adhesion of SW620 on a fibroblast layer in presence or absence of FGFR inhibitors and a FGF-2 specific blocking antibody. **C.** Adhesion of SW620 on fibroblasts in presence of EMD-121974, and specific integrin blocking antibodies as indicated. All data are represented as mean +/− SD.

These observations suggested that immobilized, rather than soluble FGF-2 might be responsible for these effects. To demonstrate that FGF-2 was localized at the cell surface, cell surface proteins were labeled with biotin, isolated by avidin precipitation and analyzed by Western blotting for FGF-2. Indeed FGF-2, but not GAPDH (a typical cytoplasmic protein) was detected in the biotinylated membrane protein fraction of fibroblasts cultures (Fig. 7A). These results demonstrate the presence of FGF-2 at the cell surface. Consistent with this observation, SW620 and HT29 adhesion on fibroblasts was strongly reduced in the presence of an anti-FGF-2 antibody and small molecular FGFR kinase inhibitors (Fig. 7B and Supplementary Fig. 5A). Furthermore, an α_V_β_3_/α_V_β_5_ integrin blocking cyclic RGD peptide, EMD-121974, and an anti-α_V_β_5_ blocking antibody, but not anti-α_V_β_6_ or anti-β_1_ blocking antibodies, strongly reduced SW620 and HT29 adhesion on fibroblasts (Fig. 7C and Supplementary Fig. 5B). Expression of α_V_β_5_ and α_V_β_6_, but not α_V_β_3_ on cancer cells, was demonstrated by flow cytometry analysis and was not altered by co-culture with cancer cells (Supplementary Fig. 5C).

From these experiments we concluded that SW620 and HT29 CRC cell adhesion to fibroblasts depends on fibroblasts cell surface associated FGF-2, FGFR signaling and α_V_β_5_ integrin ligation.

### Fibroblasts-induced cancer cells adhesion, elongation, migration and invasion require SRC activation in cancer cells

**Figure 8:**
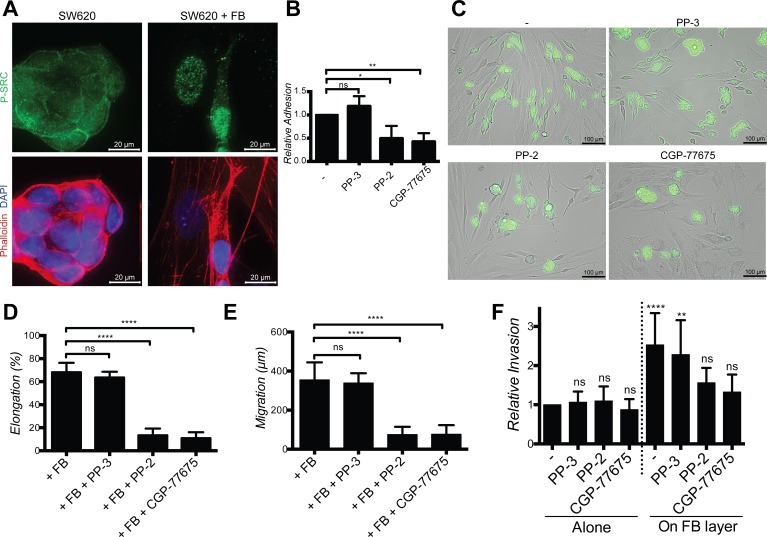
SRC in cancer cells mediate elongation, migration and invasion induced by fibroblasts **A.** Representative images of SW620 SRC activation (green) in the presence or absence of fibroblasts, stained with DAPI (blue) and Phalloïdin (red). **B.** Adhesion of SW620 on fibroblasts in presence or not of PP-3 (neg. ctrl), PP-2 and CGP-77675 SRC inhibitors. **C.** Representatives images of SW620-GFP cultured with PP-3, PP-2 and CGP-77675 in the presence of fibroblasts for 48 h. **D.** Quantification of SW620 elongation with or without SRC inhibitors. **E.** Quantification of SW620 motility with or without SRC inhibitors for 48 hours. **F.** Quantification of SW620 2D spheroid invasion as indicated after 4 days. All data are represented as mean +/− SD.

**Figure 9:**
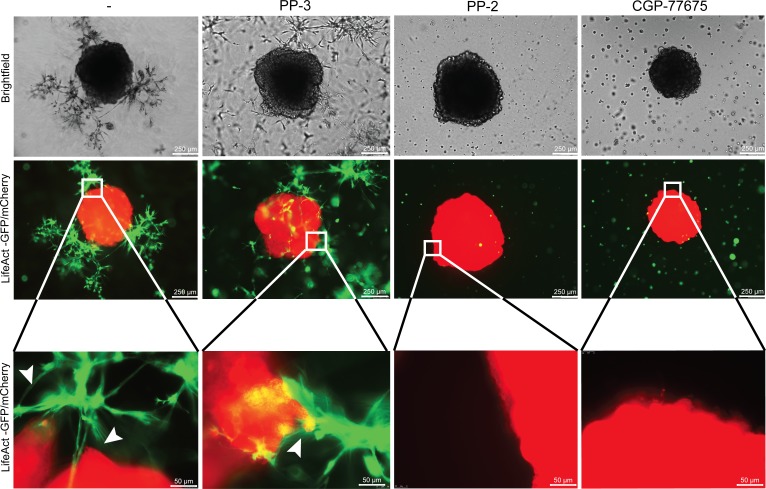
SRC in cancer cells mediate invasion induced by fibroblasts Representative images of SW620-mCherry 3D spheroid invasion after 4 days with fibroblasts-GFP cultured with PP-3, PP-2 and CGP-77675 SRC inhibitors as indicated.

Next we looked for an intracellular signaling pathway mediating FGF-2/FGFR-induced cancer cell elongation, migration and invasion. As inhibition of PI3K/AKT, MAPK/ERK, ROCK and FAK did not prevent these effects (Supplementary Fig. 4A), we focused on SRC, a kinase downstream of FGFR and integrins known to modulate cell adhesion, migration and invasion [25, 26]. Basal levels of membrane-associated phospho-SRC were observed in SW620 and HT29 cultured alone and more frequently in elongated cancer cells, in particular at sites of contact with fibroblasts. Nuclear phospho-SRC was observed in cancer cells in contact with fibroblasts and in fibroblasts themselves (Fig. 8A and Supplementary Fig. 6A). Inhibition of FGFR kinase activity fully or partially inhibited SRC phosphorylation (Supplementary Fig. 6B). Inhibition of SRC activity with PP-2 or CGP-77675 (Supplementary Fig. 6C) effectively inhibited SW620 and HT29 adhesion on fibroblasts (Fig. 8B and Supplementary Fig. 6D), fibroblasts-induced SW620 and HT29 elongation and motility during co-cultures (Fig. 8C-8E and Supplementary Fig. 6E and 6F). The related inactive compound PP-3 was ineffective to induce any effect. SRC inhibition also prevented scattering and invasion of cancer cells in the 2D and 3D spheroid models (Fig. 8F and Fig. 9). Importantly, under these experimental conditions, SRC and FGFR inhibitors were not cytotoxic (Supplementary Fig. 6G).

The use of pharmacological inhibitors in these experiments has two main limitations: inhibitors are not fully specific and may affect other SRC family members (i.e. Yes, Fyn, Fgr, Lck or Hck) and also target SRC in fibroblasts. To circumvent these limitations we silenced SRC through lentiviral-mediated expression of SRC-specific shRNA in cancer cells or in fibroblasts (Supplementary Fig. 7A). SW620 and HT29 cells with silenced SRC failed to elongate and migrate in the presence of fibroblasts (Supplementary Fig. 7B–7D). In contrast, fibroblasts with silenced SRC expression were still able to induce cancer cell elongation (Supplementary Fig. 7E and 7F).

From these experiments we concluded that fibroblast-induced cancer cell elongation, motility and invasion depend on SRC activation in cancer cells.

## DISCUSSION

**Figure 10:**
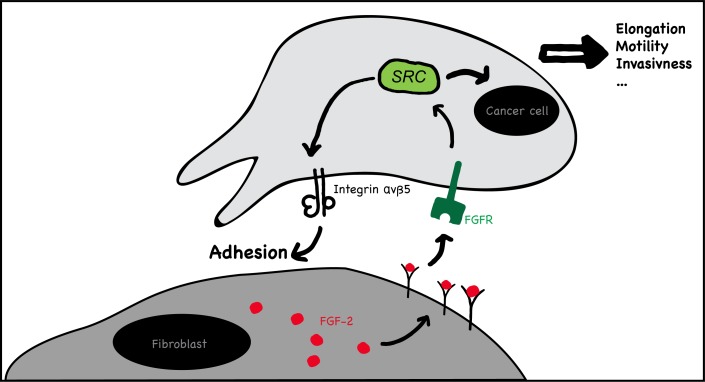
Schematic representation of the proposed mechanism of fibroblast mediated contact-dependent cancer cell migration Fibroblasts surface associated FGF-2 activates FGFR on cancer cells, which in turn activates SRC. Activated SRC induces integrin α_v_β_5_-dependent tumor cell adhesion to fibroblasts and tumor cell motility.

CAF promote cancer invasion and metastasis through the combined promotion of cancer cell growth, survival and motility, induction of angiogenesis and ECM modification [10, 15, 19]. These effects are largely considered mediated by released factors (i.e. cytokines, chemokines, angiogenic factors and matrix proteases). Considering the close physical proximity of cancer cells and CAF at CRC invading fronts [9], it appears reasonable that direct CAF-cancer cell contact may also play a role. To date, however, the role of direct CAF contact in CRC cell invasion and the putative involved mechanisms remain largely elusive. Here we addressed this question by using *in vitro* 2D and 3D co-cultures systems SW620 and HT29 CRC cells and cultured dermal, normal colon and colon cancer fibroblasts. We found that fibroblasts induce cancer cell motility and invasion in a cell-contact dependent manner and we provide a mechanism: Fibroblasts surface associated FGF-2 activates FGFR on cancer cells, which in turn activates SRC. Activated SRC induces integrin α_v_β_5_-dependent tumor cell adhesion to fibroblasts and tumor cell motility (Fig. 10). In 2D models, cancer cells migrate along fibroblasts while in the 3D models fibroblasts “pull” cancer cells to initiate and guide migration into the gel.

The relevance of our results relies on the following implications. *Firstly*, they demonstrate the functional importance of direct fibroblast-cancer cell contact. *In vivo* this may be especially important under conditions of migration along precise paths, such as matrix fibers [27], or when cancer cells have not yet acquired full invasive capacities, for instance during invasion in the absence of EMT [28]. To corroborate this hypothesis additional experiments using several cancer cell lines with different degree of malignancy and *in vivo* experiments are necessary. *Secondly*, they provide a new role for fibroblasts cell surface-associated FGF-2. The role of FGF-2 in cancer progression is well recognized [29, 30]. While it is well known that FGF can associate to the cell surface through its high affinity for glycosaminoglycans [31], the biological significance of cell surface FGF has not been well characterized. Rather, its biological activities have been attributed to the soluble and matrix-associated fraction [29]. Our observations suggest that FGF-2 may contribute to distinct phases of cell invasion: cell surface associated FGF-2 may initiate migration of poorly invasive cells requiring additional cell-derived stimuli, while matrix-associated FGF-2 might enhance invasion of already invasive cells. The exact process of FGF-2 presentation at the cell surface leading to these effects remains unknown at this point and its elucidation will require additional work. *Thirdly*, we identify FGFR, SRC and α_v_β_5_ as the molecules mediating cancer cell adhesion on fibroblasts, migration and invasion. To our surprise, α_v_β_6_, an integrin up-regulated in CRC (and present on CRC cells) and contributing to CRC invasiveness through the activation of TGF-β and production of MMPs [32], was not involved in this effect. How FGFR, SRC and α_v_β_5_ interact in this context, in particular whether activated FGFR associate with α_v_β_5_ integrin, remains still unknown. As β_5_ integrin and FGFR1 are reported to promote CRC progression [33, 34], we performed co-immunoprecipitation experiments to test whether these receptors may associate upon FGF-2 presentation. However, we could not demonstrate co-precipitation of β_5_ integrin and FGFR1 under mild detergent conditions and complex stabilization by surface protein-protein cross-linking (Supplementary Fig. 8). Although preliminary, these results suggest that either FGFR1 is not the receptor of interest for this effect, among the FGFR family members, or that indeed FGFR1 and α_v_β_5_ do not associate. Additional experiments are necessary to clarify this point. The exact role of SRC signaling and nuclear translocation remains also open. Preliminary observations indicate that SRC is not regulating α_v_β_5_ activity through direct phosphorylation of the β_5_ cytoplasmic domain (data not shown). A further important open question concerns the α_v_β_5_ ligand on the fibroblast surface. As FGF-2 was reported to bind to α_v_β_3_ [35], we tested whether SW620 and HT29 cells adhered to immobilized FGF in a α_v_β_5_-dependent manner. While cancer cells adhered to immobilized FGF-2 this adhesion was not integrin dependent (data not shown). We also tested the potential role of heparin, which is a necessary binding-partner for FGF-2 in order to stabilize receptor binding and dimerization. However, results suggest that heparin is not playing a detectable role in promoting/perturbing cancer cell adhesion to fibroblasts (data not shown). A role of fibroblast surface fibronectin, as a possible α_v_β_5_ ligand for cancer cell adhesion, was also excluded using anti-fibronectin-adhesion blocking antibodies (data not shown). Further, N-cadherin, which was reported to promote cancer cell invasion and metastasis in association with FGFR1 [36] and fibroblasts, was also excluded as not expressed in these cancer cells (data not shown). We are now considering additional candidate ligands. *Fourthly*, as inhibitors of FGFs, FGFRs, SRC and α_v_ integrins are in preclinical development or already available for clinical testing [37–40], these results provide a rationale for *in vivo* preclinical testing and focusing on their clinical assessment in invasive CRC. To assess the clinical relevance of these *in vitro* observations, we performed preliminary immunohistochemical stainings for FGF-2 and FGFR1 on human colorectal cancer samples. Representative results, shown in Supplementary Fig. 9, confirm the presence of FGF-2 in both cancer cells and stromal cells. FGFR1 staining is present on more differentiate areas of the cancer, but lost in more transformed, and invasive regions. This is consistent with concurrent observations suggesting that FGFR1 is not the major receptor involved in this process. Membrane localization on fibroblasts cannot be confirmed in these stainings. *Fifthly*, they raise a fundamental question on the role of fibroblasts in cancer cell invasion: which cues do cancer cells gain from adhering on fibroblasts to initiate elongation and migration, that they cannot acquire through fibroblasts-released factors and deposited matrix? Initial experiments addressing this question indicate that cell contact induces important changes in gene expression in both fibroblasts and cancer cells.

A corollary of this study is the observation that fibroblasts cultured and expanded *in vitro* have similar gene expression profiles and activation markers independently on the *in vivo* origin, including for those originally from the tumor stroma. These observations, in combination with the results from laser micro-dissected stroma, suggest that comparative studies of CAF *vs*. normal fibroblasts are best performed using freshly isolated, non-cultured, cells.

In conclusion, we show that fibroblasts induce cell-contact-dependent CRC cell migration and invasion under 2D and 3D conditions *in vitro* through surface associated FGF-2, FGFR-mediated activation of the SRC in cancer cells and α_v_β_5_-mediated adhesion. Further characterization of this effect may reveal novel aspects on the cancer invasion-promoting role of fibroblasts.

## MATERIALS AND METHODS

### Cell culture

The human colorectal carcinoma cell lines SW620 and HT29, and HEK-293T cells were purchased from ATCC (LGC Standards). CAF, dermal and colon fibroblasts and Human Umbilical Vein Endothelial Cells (HUVEC) were obtained from human samples. SW620 were cultured in RPMI GlutaMAX™, HT29, all fibroblasts and HEK-293T in DMEM GlutaMAX™, all supplemented with 10% FCS, 100 U/ml Penicillin and 100 μg/ml Streptomycin. HUVECs were cultured as described previously [41]. All cell culture reagents were purchased from Life Technologies.

For co-culture experiments, fibroblasts and colon cancer cells were grown for 48 hours at a 1:1 ratio in DMEM GlutaMAX™. Conditioned media were harvested after 72 hours of culture and filter 0.45 μM before use. Coatings were performed over-night using porcine Gelatin 0.5% (Sigma), Poly-L-Lysine 0.1% (Life Technologies), human Fibronectin 0.1% (Sigma) and 1% Rat Tail Collagen Type I (BD Bioscience) in PBS. TGF-β (Sigma) was used at 20 ng/ml, EGF and FGF-2 (Cell Signaling) at 100 pg/ml. Cell counting and viability determination was performed by trypan blue exclusion using Neubauer Counting Chamber.

To produce conditioned matrix, fibroblasts were grown to confluence for 3 days, and then detached over-night at 4°C using 20 mM EDTA (Sigma), 10 μg/ml Leupeptin (Sigma), 1 mM PMSF (Sigma) and 10 μg/ml Soybean (Sigma). For fibroblast layer fixation, 4% PFA (Electron Microscopy Sciences) was applied for 2 minutes followed by washing with PBS.

Inhibitors were used at the indicated concentrations and blocking antibodies at 10 μg/ml, all added to the serum free medium and incubated with cancer cells and fibroblasts for 48 hours.

### Inhibitors of signaling

Y-27632 (Sigma), PP-3 and PP-2 (ABCAM) were used at 10 μM, Ly-294002 (Sigma) and PD-98059 (Enzo Life Sciences Inc.) at 20 μM, CGP-77675 (Sigma) at 5 μM, SU-11274 (Sigma) at 2 μM, Wortmannin (Sigma) at 0.1 μM, AG-1478 (Merck Millipore) at 200 nM, PF-562271 (Pfizer), PD-173074 (Tocris), EMD-121974 (Medkoo Biosciences), PF-573228 and PD-161570 (Sigma) at 1 μM.

### Antibodies

The following antibodies against human were used for Western blotting and immunoprecipitation technic: Anti-GAPDH (Sigma), anti-phospho-SRC Tyr416 and anti-SRC (Cell Signaling), anti-FGF-2 (Thermo Fisher Scientific Inc.), anti-FGFR1 and anti-β_5_ (Cell Signaling), all at 1/1000 dilution. Secondary antibodies used were: anti-rabbit-HRP and anti-mouse-HRP (Dako), both at 1/1000 dilution.

For FACS analysis, anti-α_v_-PE and anti-β_5_ (Biolegend), anti-β_3_-PE and anti-α_v_β_3_-PE (BD Biosciences), anti-β_6_ (clone IC8C3, Dr. D. Sheppard, UCSF, San Francisco) were used at 1/100 dilution. Anti-α_v_β_5_ (clone P5H9, R&D Systems) and anti-α_v_β_6_ (clone 10D5, Merck Millipore) were used for FACS at 1/50 dilution, and for function blocking experiments at 10 μg/ml, as well as anti-FGF-2 (R&D Systems) and anti-β_1_ (clone Lia1/2, Beckman). Dead Cell Apoptosis Kit for PI-Annexin V staining was from Life technologies.

Anti-phospho-SRC Tyr416 (Cell Signaling), anti-FGFR1 (Cell Signaling), anti-Phalloïdin-AlexaFluor 546 (Invitrogen), anti-FGF-2 (Thermo Fisher Scientific Inc.), as well as anti-αSMA (Sigma), anti-pan-Cytokeratin (Dako) and DAPI ProLong Gold mounting medium (Invitrogen) were used for immunofluorescent staining and immunohistochemistry.

### Co-culture separation

Cancer cells were separated from fibroblasts using MACS^®^ separation technique, following manufacturer's instructions. MS columns and anti- human Fibroblast micro-beads were used. All reagents were from Miltenyi Biotec.

### Isolation of fibroblasts and HUVEC

Dermal fibroblasts were isolated from human neonatal foreskin. Excised skin samples were was placed into Ca^2+^, Mg^2+^-free Hank's balanced salt solution (Life Technologies) with 100 U/ml Penicillin-Streptomycin (Life Technologies) and 5 μg/ml Gentamicin (Sigma). After removal of subcutaneous fat, skin samples were incubated 2 hours at 37°C with 2–4 ml of collagenase type I 1 mg/ml (Roche). Cell suspension was harvested on ice, quenched by adding DMEM and filtered 70 μM. 2′000′000 cells per dish were plated with DMEM. The medium was replaced twice a week and cells maintained up to 80% confluence. Dermal fibroblasts were used up to 15 passages.

Colon fibroblasts and CAF were isolated according to the protocol by Orimo et al. [21]. Tissue sections, obtained following colectomy for colon cancer, of about 1 cm^3^ were collected in 15 ml DMEM, transported on ice and washed about 5 times with DMEM, before being cut into small pieces, washed again and incubated for 12 h at 37°C with agitation in 2–4 ml DMEM containing collagenase type I 1 mg/ml and 120 units hyaluronidase (Sigma). The dissociated tissues were incubated without shaking for 5 minutes at room temperature, followed by the separation of stromal cell-enriched supernatant to a new tube. The stromal fraction was centrifuged and cells were cultured on tissue culture plates in DMEM for 15–20 cell passages.

HUVEC were isolated as previously described [42] from fresh umbilical cords dissociation.

Protocols for collection and use of human samples were approved by the Ethic committees of Cantons Vaud, Berne and Ticino, Switzerland.

### Spheroids assay

Cancer cells spheroids were prepared as previously described [43], using the hanging drop technic in a Terazaki plate with 500 cells per well in 20 μl medium for 72 h. 3D spheroids assay were performed according to the 3D-On-Top method [44]. 7 mg/ml Matrigel growth factors reduced (Corning) was used as matrix, mixed or not with 75’000 fibroblasts/ml. For 2D spheroids assay, cancer cell spheroids were placed on top of a confluent fibroblasts layer or on coated wells.

### Vectors and infections

LifeAct lentiviral vectors in GFP and mCherry were kindly provided by Dr. Olivier Pertz. pSD44 lenti-vector expressing GFP and DsRED were pRRLSIN.cPPT.PGK/GFP.WPRE lentiviral vector under the control of the phospho glycerate kinase promoter. SRC pLKO.1 shRNA were from Thermo Fisher Scientific Inc. and used following manufacturer's instructions.

Lentiviral particles were generated in HEK-293T cells by transducing the vector of interest with pMD2G (pSD11) and pMDLgpRRE (pSD16) plasmids using calcium phosphate transfection [45], followed, depending of the vector used, by an antibiotic-based selection of the infected cells.

### Western blotting

Cells were lysed using RIPA lysis buffer (Cell Signaling) supplemented with protease Cocktail inhibitor (Sigma), 2 mM PMSF, 2 mM BGE (Sigma) and 0.2 mM Orthovanadate (Sigma) on ice and re-suspended in SDS buffer containing 10% Glycerol, 5% β-mercatoethanol, 60 mM Tris-Cl, trace of bromophenol blue and 2% SDS, all from Sigma. SDS-PAGE, blotting, and detection were performed as previously described [46].

Cell Surface Protein Isolation Kit (Pierce) was used for isolation of membrane proteins fraction following manufacturer's instructions.

### Co-Immunoprecipitation

Cells were lysed using RIPA non-denaturant lysis buffer (Cell Signaling) supplemented with protease Cocktail inhibitor, 2 mM PMSF, 2 mM BGE and 0.2 mM Orthovanadate on ice. After sonication and centrifugation the supernatant was incubated for 12 hours at 4°C under agitation with primary antibody at 1/50 dilution. The mix was then incubate for 30 minutes at 4°C with protein A magnetic beads (Cell Signaling), followed by beads isolation using magnetic separation rack (Cell Signaling). Samples was heated for 5 minutes at 95°C, mixed with SDS buffer and processed by SDS-PAGE and Western blotting.

For protein cross-linking experiments, cells were treated for 30 minutes at room temperature with 2 mM DSP (Thermo Fisher Scientific Inc.) followed by 15 minutes quenching using 1 M Tris pH 7.5. Cells were lysed and processed as above.

### ELISA

For FGF-2 quantification, ELISA assays (Biolegend) was performed following manufacturer's instructions. For fibronectin detection, fibroblasts were removed from cell culture plates as previously described, wells were blocked using 5% BSA and incubated with anti-fibronectin antibody (Sigma), followed by a goat-anti-mouse-HRP antibody and a chromogenic substrate (Biolegend). Absorption was measured at 630 nm and normalized comparing fibronectin coated-wells.

### Flow cytometry

Annexin V/PI staining was performed following manufacturer's instructions (Life Technologies). Integrin staining were performed using directly labeled anti-integrin primary antibodies 1:100 dilution 30 minutes on ice or unlabeled primary antibodies followed by 30 minutes secondary antibody anti-rabbit or mouse-PE (BD Bioscience) incubation on ice (1:100 dilution). PBS with 3% FCS and 5 mM EDTA was used as buffer. BD FACSCalibur (Becton Dickinson) instrument was used to perform experiments and FlowJo 9.7.4 (Treestar Inc.) software was used to analyze all data.

### Adhesion assay

8’000 fibroblasts were cultured in a 96-wells plate to reach confluence. 15’000 GFP-cancer cells were added on fibroblasts layer and incubated in serum free medium for 3 hours. Non-adherent cells were removed using ice cold PBS and adherent cells were observed and quantified using fluorescence microscopy.

### Immunostaining

Cells were grown on a glass chamber-slide previously coated with Poly-L-lysine followed by 10 minutes fixation with 4% PFA, 5 minutes permeabilisation with 0.1% Triton X-100 (Sigma) in PBS and blocking for 30 minutes with PBS containing 0.05% BSA, 5% Donkey serum (Fitzgerald) and 0.1% Triton X-100. Primary antibody was incubated over-night at 4°C at 1/100 dilution. Secondary and third antibodies were incubated for 1 hour at room temperature in blocking at 1/100 dilution. DAPI staining was included in the mounting medium.

For immunohistochemistry, paraffin embedded organ cuts were pre-treated 30 minutes with Citrate (Leica) for FGF-2 and FGFR1 stainings, or 15 minutes in Enzyme (Leica) for Cytokeratin staining. Primary antibodies were incubated for 30 minutes at the following dilutions: FGF-2, 1:25; FGFR1, 1:50; Cytokeratin, 1:200; αSMA, 1:8000. Slide stainings were performed on Leica BOND RX system following manufacturer's instructions.

### RT-PCR

mRNA was extracted using RNeasy Mini kit (Qiagen) and total RNA was retro-transcribed using Superscript following manufacturer's instructions (Promega AG). cDNA was subjected to PCR amplifications using the following primer pairs (Eurofins MWG Operon) at the indicated hybridization temperatures. Real-time qPCR was performed using the StepOne SYBR System (Life Technologies). GAPDH 58°C (Fw-TCTTCTTTTGCGTCGCCAGC, Rev-GATTTTGGAGGGATCTCGCTCCT), α-SMA 58°C (Fw-AGGAAGGACCTCTATGCTAACAAT, Rev-AACACATAGGTAACGAGTCAGAGC), FAP 62°C (Fw-CAAGTGGCAAGTGGGAGGCCA, Rev-TGGGGATGCCTGGGCCGTAG), SDF-1 62°C (Fw-TGAGCTACAGATGCCCATGC, Rev-TTCTCCAGGTACTCCTGAATCC), IL-6 60°C (Fw-TCGAGCCCACCGGGAACGAAA, Rev-GACCGAAGGCGCTTGTGGAGA), VIM 62°C (Fw-GAAGGCGAGGAGAGCAGGATTTC, Rev-AGTGGGTATCAACCAGAGGGAGTG), FSP-1 58°C (Fw-TTGGGGAAAAGGACAGATGAAG, Rev-TGAAGGAGCCAGGGTGGAAAAA), FGF-2 60°C (Fw-CGCCCGGCCACTTCAAGGAC, Rev-AGCTTGATGTGAGGGTCGCTCTTC)

### Gene expression analysis by microarray hybridization

Laser capture micro-dissections were performed as described in the paper of Farmer et al. [47]. RNA extraction, micro-array and data analysis were performed as described in the paper of Christensen et al. [48]. The complete data set is publicly available at GEO (http://www.ncbi.nlm.nih.gov/geo/) through the accession number GSE30292 and GSE23583.

Robust multi-array averaging (RMA) and quantile normalization were used to quantify gene expression, for non-supervised hierarchical clustering Pearson's correlation and average linkage clustering, respectively, were used for similarity measurement and clustering using R (R 2.12.0, Bioconductor 2.7). Principle component analysis was performed using R 3.1.

### Gene expression analysis by RNASeq

All fibroblasts and HUVEC cells were grown at the same time in triplicate from independent patients at passage 6. Total RNA was extracted using the total RNA extraction Nucleospin II kit (Machery-Nagel). The quality and quantity of all RNA samples were examined by Agilent 2100 Bioanalyzer (Agilent Biotechnologies) and NanoDrop (Witec AG).

RNA-seq libraries were prepared using 15 ng of total RNA and the Ovation Nugen RNA-Seq System v2 kit (Nugen). 100 ng of the ds-cDNAs obtained were fragmented to 350 pb using Covaris S2 (Covaris). The Illumina TruSeq nano reagents (Illumina) were used to generate the final libraries. Cluster generation was performed with the resulting libraries using the Illumina HiSeq PE Cluster Kit v4 reagents and sequenced on the Illumina HiSeq 2500 using HiSeq SBS Kit v4 reagents. Sequencing data were processed using the Illumina Pipeline Software version 1.82.

Purity-filtered reads were adapters and quality trimmed with Cutadapt (v.1.3) and filtered for low complexity with seq_crumbs (v.0.1.8). Reads were aligned against *Homo sapiens.GRCh38.76* genome using STAR (v.2.4.0f1) [49]. The number of read counts per gene locus was summarized with htseq-count (v.0.6.1) [50] using *Homo sapiens.GRCh38.76* gene annotation. Quality of the RNA-seq data alignment was assessed using RSeQC (v.2.3.7) [51]. Reads were also aligned to the *Homo sapiens.GRCh38.76* transcriptome using STAR and the estimation of the isoforms abundance was computed using RSEM (v.1.2.16) [52]. RSEM counts were used for further analysis. Principle component analysis was performed using log2 transformed RSEM counts (R 3.1). Heatmaps were generated using package gplots in R 3.1. The complete data set is publicly available at GEO through the accession number GSE67945.

### Imaging and data analysis

For co-culture experiments images were taken every hour at different magnifications with an inverted fluorescence microscope (Leica AF6000). Cancer cell elongation was quantified manually by scoring individual cells using Image J “Cell Counter” plugin on taken images at 48 h co-culture. Elongated cell were defined as single cell with a minimal length-width rapport of 2:1, as well as at least one sharp-ending extremity. Cancer cells motility was quantified based on single cell track follow on 24 h microscope movies using Image J “Manual Tracking” and “Chemotaxis Tool” plugins.

Imaging of 2D and 3D spheroids assay were performed with 5× and 40× objectives with Leica AF6000 inverted fluorescence microscope. Invasion was quantified using Photoshop CS6 (Adobe) by calculating the invaded area on the 4 days image, normalized on initial spheroid size.

Western Blot images were quantified for protein level using Image J “Gel Analyze Tool” plugin.

For immunostaining images were taken using a DeltaVision Elite Microscope (GE Healthcare) with 63× and 100× objectives, followed by deconvolution treatment.

For immunohistochemistry images, slides were scanned using a NanoZoomer 2.0 HT (Hamamatsu) and images were extracted using NDP.view2 analysis software (Hamamatsu).

### Statistical analysis

Each experiment was repeated independently a minimum of 3 times in triplicate conditions. Acquired data were analyzed using Prism Software (GraphPad). Statistical comparisons were performed by un-paired two-tailed Student's t test or by two-way ANOVA with Bonferroni pos*t*-test. Results were considered to be significantly from *p* < 0.05. In the figures the various *p* values thresholds are presented as follow: ≤0.05 = *, ≤0.01 = **, ≤0.001 = ***, ≤0.0001 = ****.

## SUPPLEMENTARY FIGURES


