# Supplementary material for: Fibroblast surface-associated FGF-2 promotes contact-dependent colorectal cancer cell migration and invasion through FGFR-SRC signaling and integrin αvβ5-mediated adhesion
Source: Oncotarget. 2015 May 5;6(16):14300–17. doi: 10.18632/oncotarget.3883 (PMC4546468; doi:10.18632/oncotarget.3883)
Supplement: Supplementary file 1 [file oncotarget-06-14300-s001.pdf]

SUPPLEMENTARY FIGURES

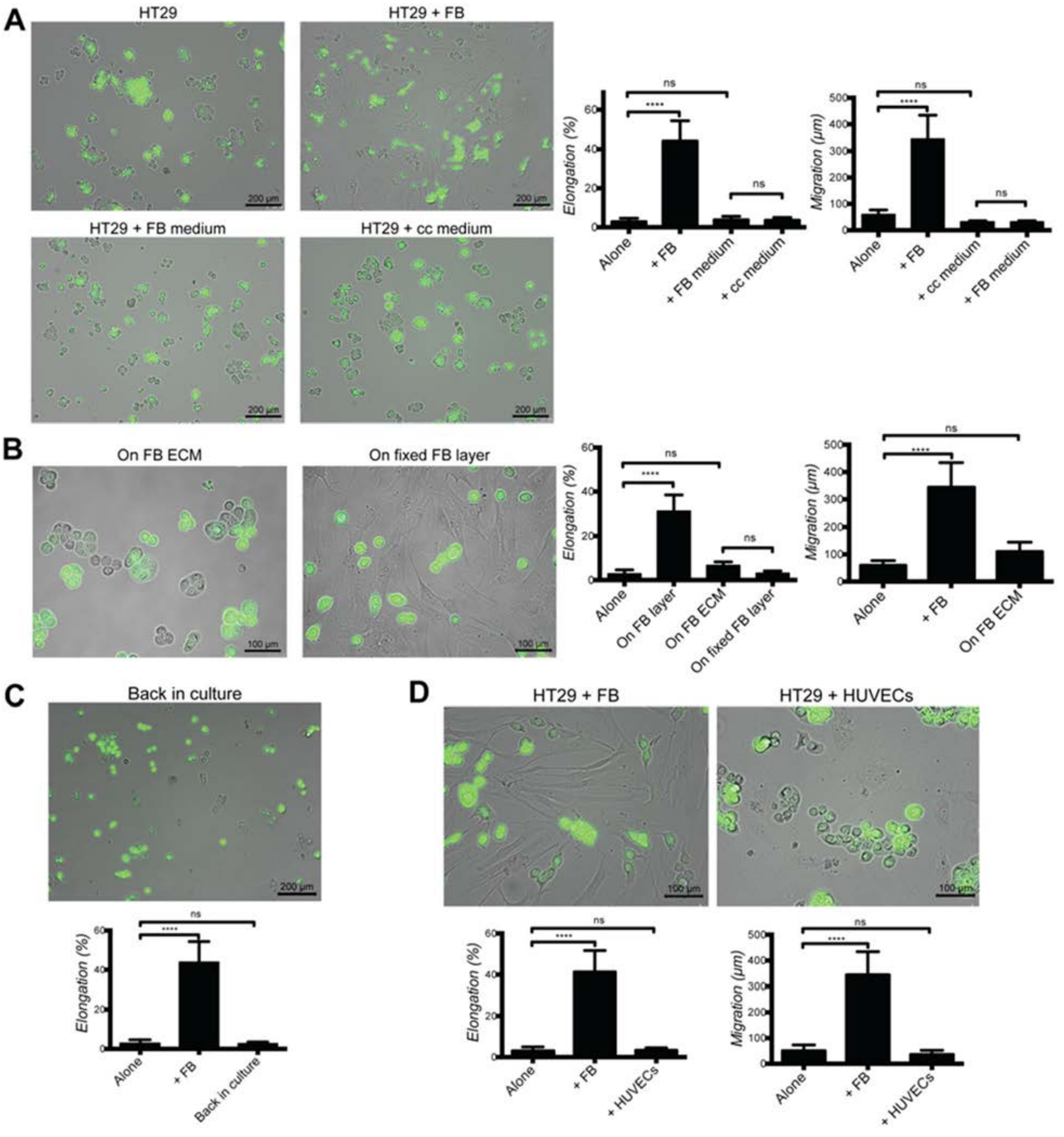

Supplementary Figure S1: Fibroblast-induced elongation of cancer cells requires direct contact with living fibroblasts. A. Representative images of HT29-GFP cultured in the presence or absence of fibroblasts, fibroblasts and co-culture (cc) conditioned media. Bar graphs represent quantification of HT29 elongation and motility during 48 hours. **B.** Representative images of HT29-GFP cultured on ECM deposited by fibroblasts and on a fixed fibroblast layer. Bar graphs represent quantification of HT29 cell elongation and motility during 48 hours. **C.** Representative image and quantification of elongation of HT29-GFP returned to culture after separation from established co-culture. **D.** Representative images and quantification of elongation and motility of HT29-GFP cultured with HUVECs and fibroblasts for 48 h. All data are represented as mean +/− SD.

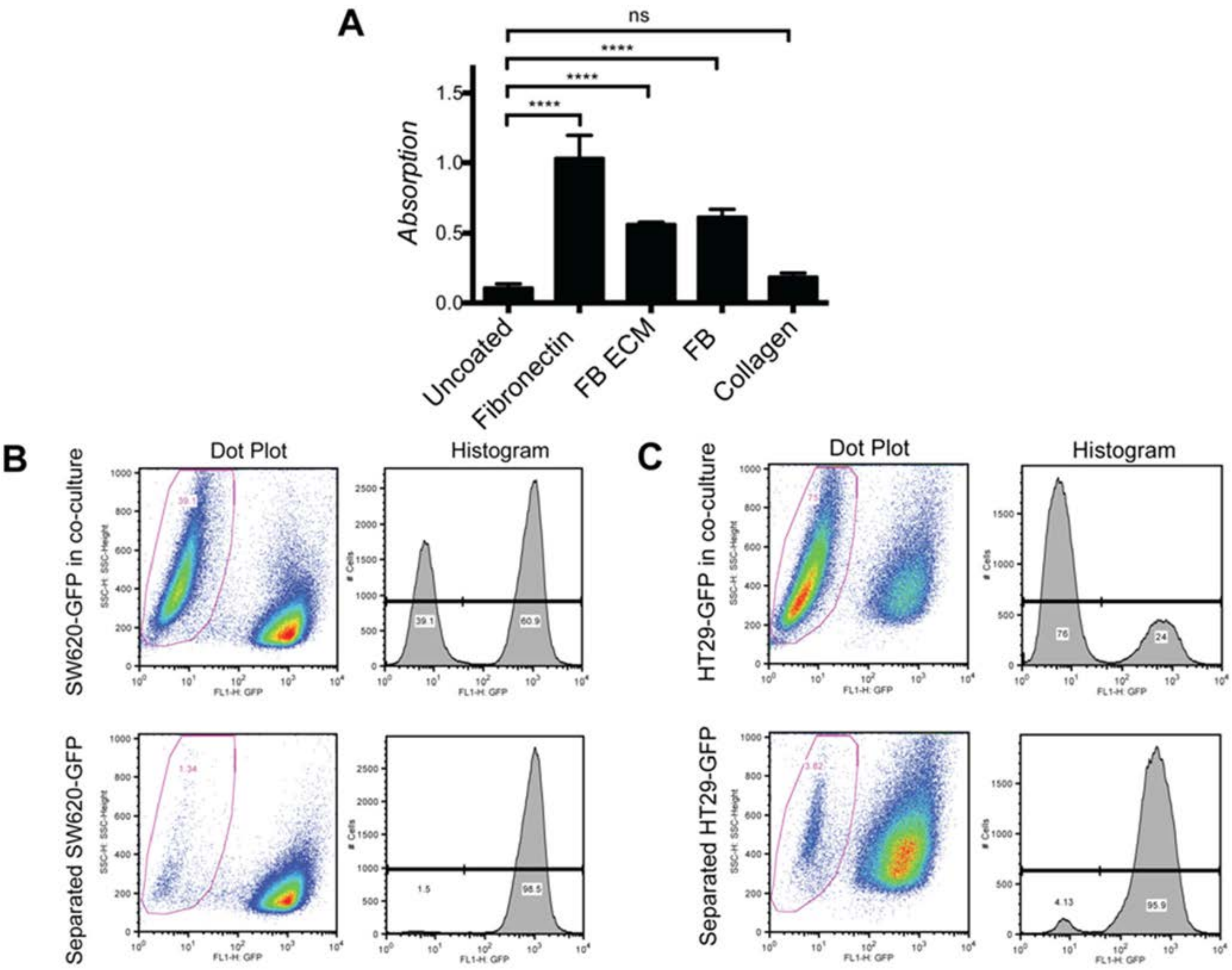

Supplementary Figure S2: Validation of isolation technics. A. Validation of fibroblasts-mediated ECM deposition by ELISA-mediated detection of fibronectin following fibroblasts removal. Absorption was normalized on fibronectin coating and represented +/− SD. **B.** FACS Analysis of SW620-GFP cultured with fibroblasts for 48 hours +/− MACS isolation of cancer cells. **C.** FACS Analysis of HT29-GFP cultured with fibroblasts for 48 hours +/− MACS isolation of cancer cells.

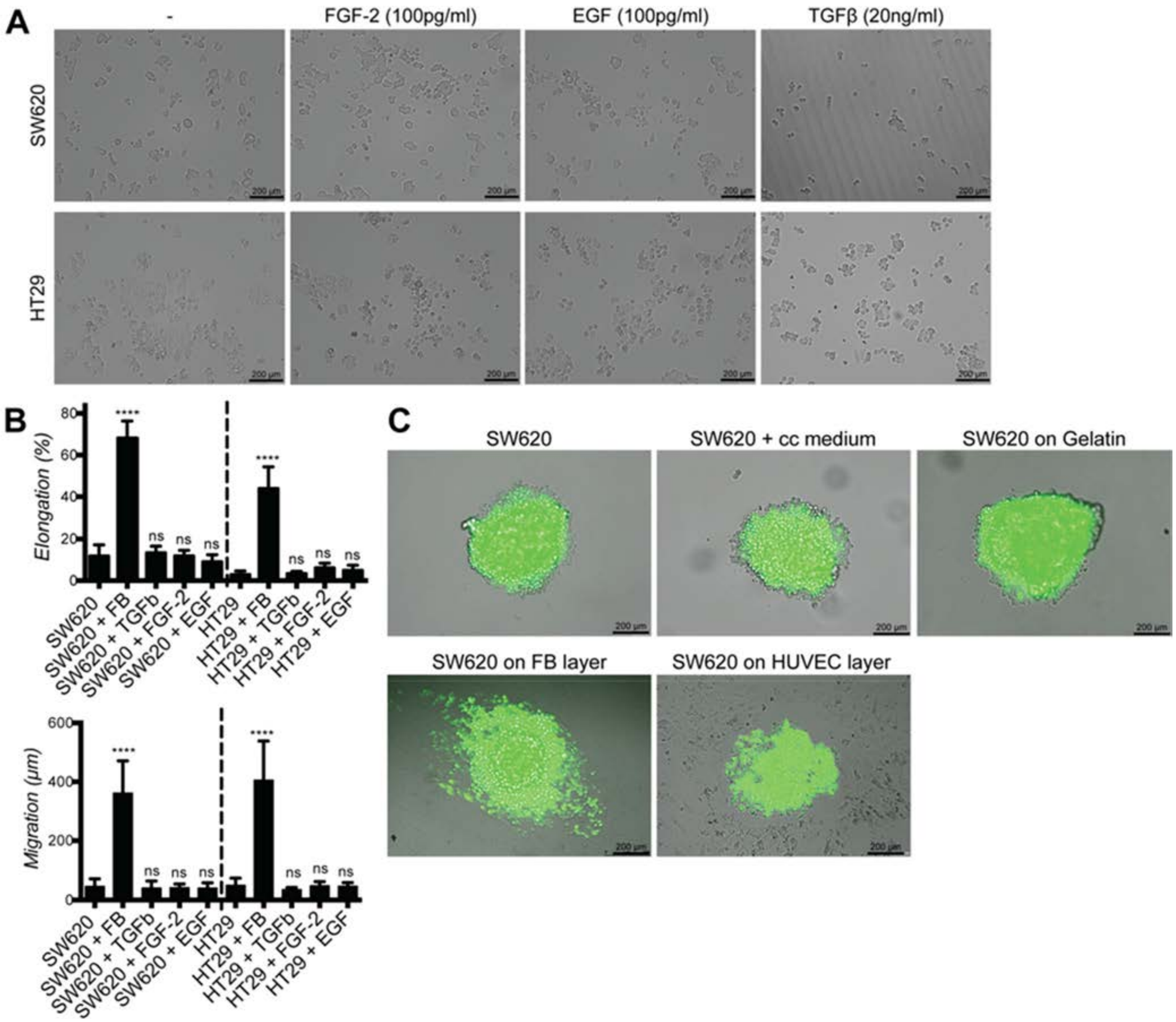

Supplementary Figure S3: Cancer cell elongation, motility and invasion abilities are not mediated by soluble factors. **A.** Representative images of SW620 and HT29 cancer cells cultured with various cytokines: TGFβ, EGF and FGF-2. **B.** Quantification of cancer cell elongation and motility during 48 hours, represented as mean +/− SD. **C.** Representative images of SW620-GFP spheroid invasion on a fibroblast layer, with co-culture (cc) medium, with gelatin coating and on HUVECs layer after 4 days.

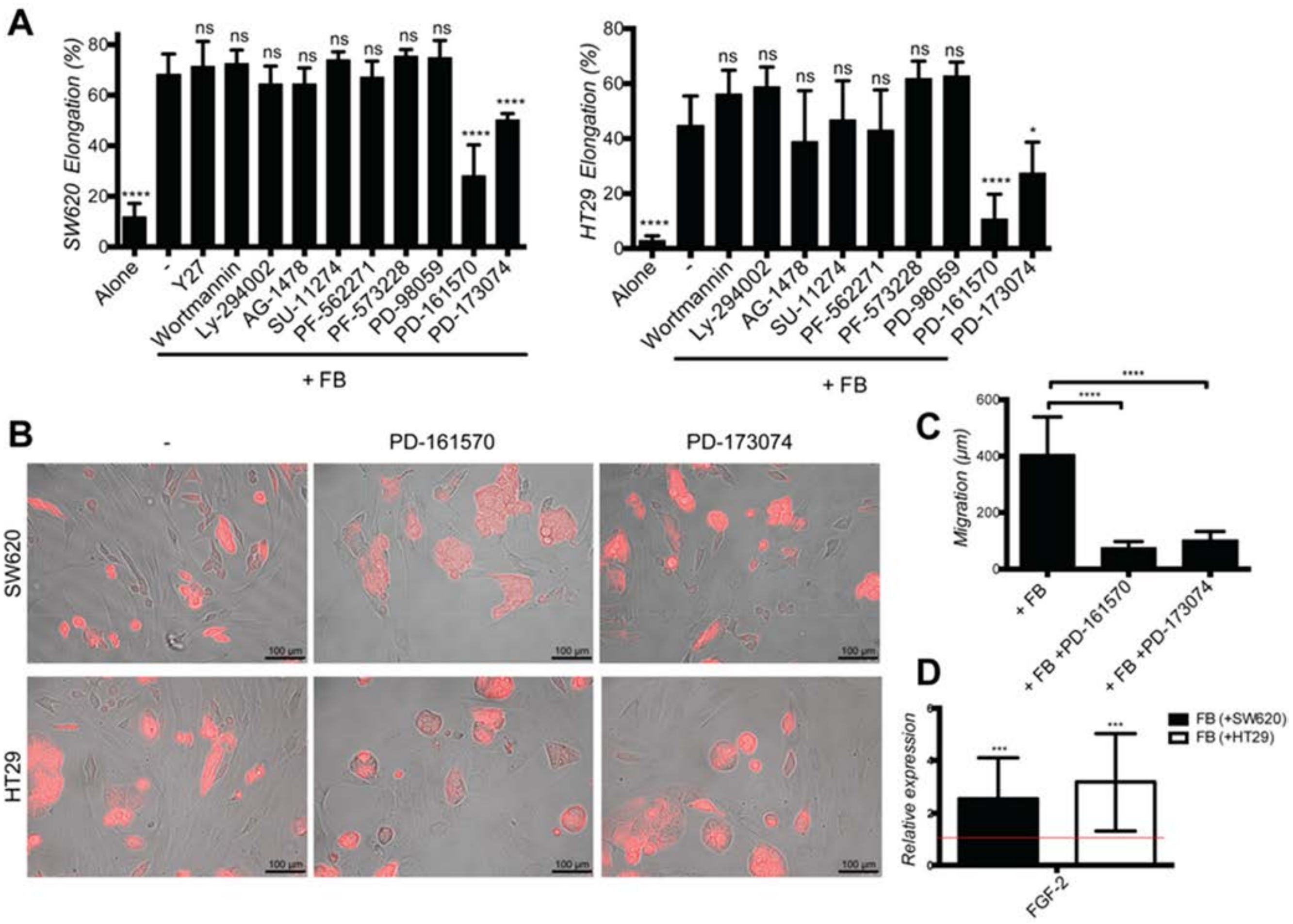

Supplementary Figure S4: Fibroblasts-mediated effects on cancer cells are mediated by FGFR. A. Quantification of SW620 and HT29 elongation in the presence of various inhibitors: Y-27632 (ROCK inhibitor), Wortmannin and Ly-294002 (PI3K/Akt inhibitors), AG-1478 (EGFR inhibitor), SU-11274 (c-Met inhibitor), PD-98059 (MAPK/ERK inhibitor), PF-562271 and PF-573228 (FAK inhibitors), PD-161570 and PD-173074 (FGFR inhibitors). **B.** Representative images of cancer cells expressing LifeAct-mCherry in co-culture with fibroblasts in presence or not of FGFR inhibitors. **C.** Quantification of HT29 motility with FGFR inhibitors in presence of fibroblasts for 48 hours. **D.** Quantification of FGF-2 mRNA variation in fibroblasts after co-culture. All data are represented as mean +/− SD.

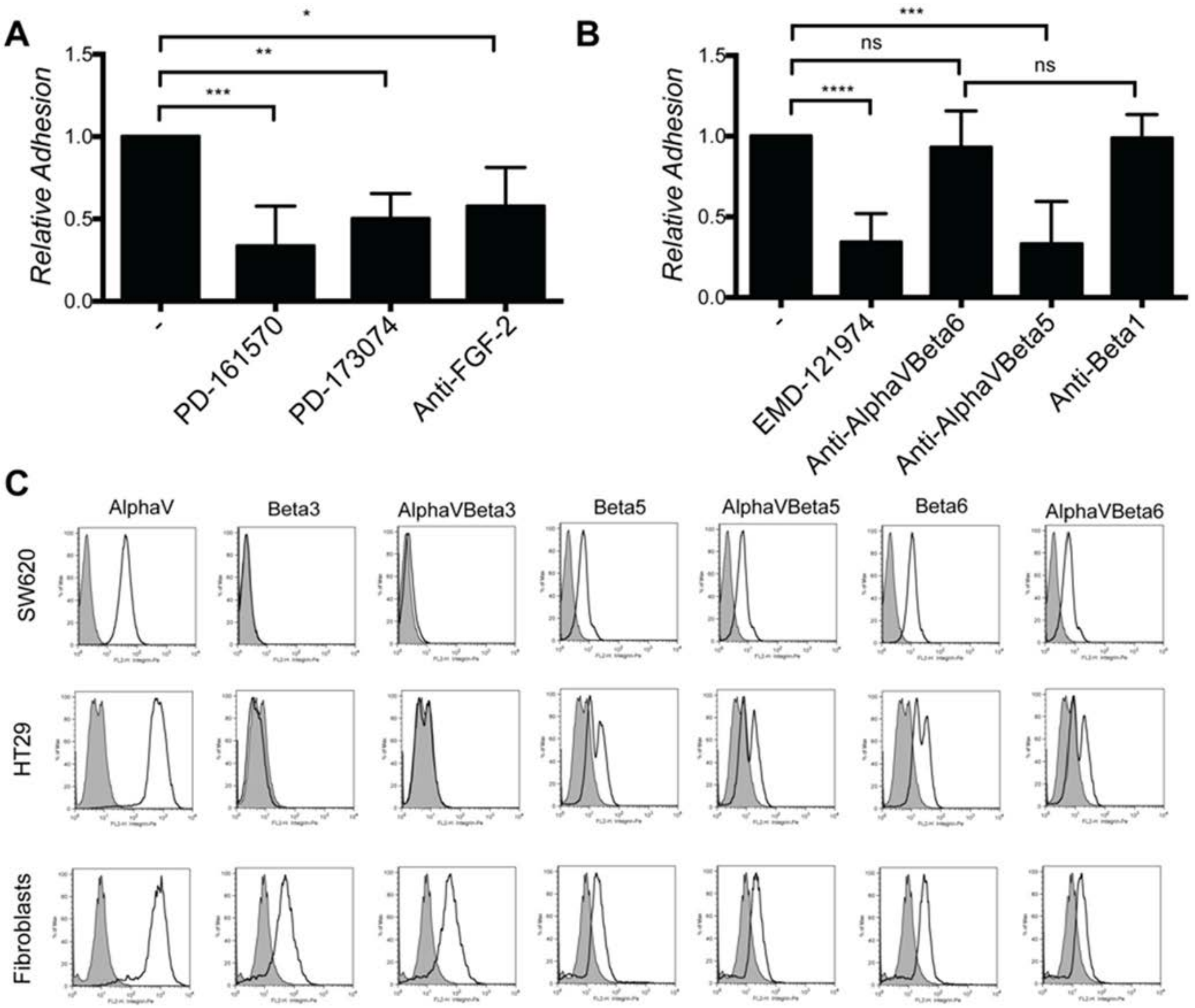

Supplementary Figure S5: Fibroblast cell surface FGF-2, FGFR and $\alpha_v\beta_5$ integrin are required for HT29 cell adhesion to fibroblasts. **A.** Adhesion of HT29 on a fibroblast layer in presence or absence of FGFR inhibitors and FGF-2 specific blocking antibody, represented as mean +/− SD. **B.** Adhesion of HT29 on fibroblasts in presence of EMD-221975, $\alpha_v\beta_6$, $\alpha_v\beta_5$ and β_1 anti-integrin specific blocking antibodies as indicated, represented as mean +/− SD. **C.** FACS histogram analysis of SW620, HT29 and fibroblast cell surface integrin expression.

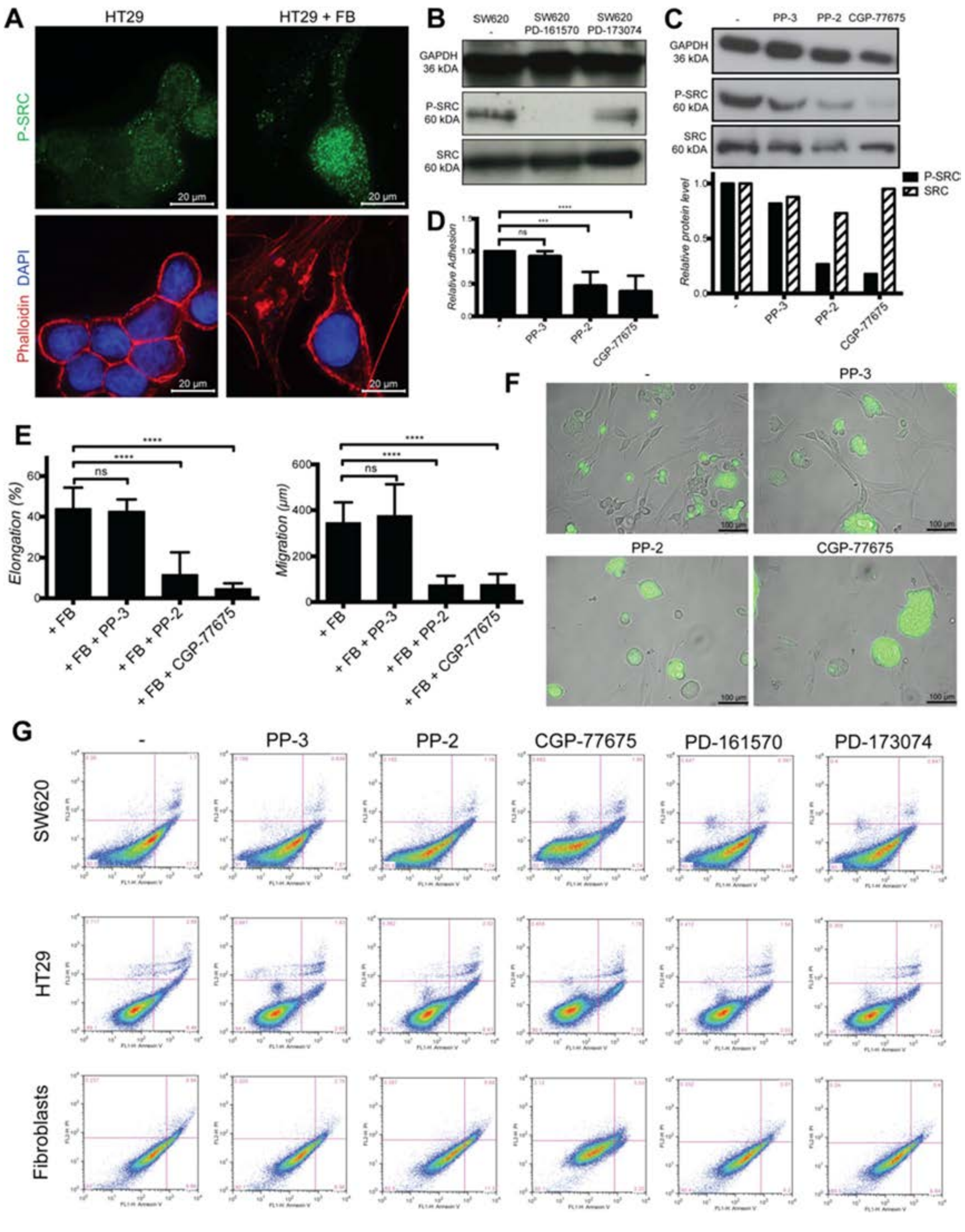

Supplementary Figure S6: SRC in cancer cells mediate cell elongation, migration and invasion induced by fibroblasts. **A.** Representative images of HT29 SRC activation (green) in the presence or absence of fibroblasts, stained with DAPI (blue) and Phalloidin (red). **B.** Western Blot quantification of SRC activity in SW620 in presence of FGFR inhibitors. **C.** Western Blot validation of SRC activity down-regulation with PP-3 (neg. ctrl), PP-2 and CGP-77675 SRC inhibitors. **D.** Adhesion of HT29 on fibroblasts in presence or absence of SRC inhibitors, represented as mean +/− SD. **E.** Quantification of HT29 elongation and motility with PP-3, PP-2 and CGP-77675 in presence of fibroblasts for 48 hours represented as mean +/− SD. **F.** Representatives images of HT29-GFP cultured in the same conditions at 20× magnification. **G.** Viability staining using FACS for SW620, HT29 and fibroblasts cultured with DMSO, PP-3, PP-2 and CGP-77675 SRC inhibitors, PD-161570 and PD-173074 FGFR inhibitors.

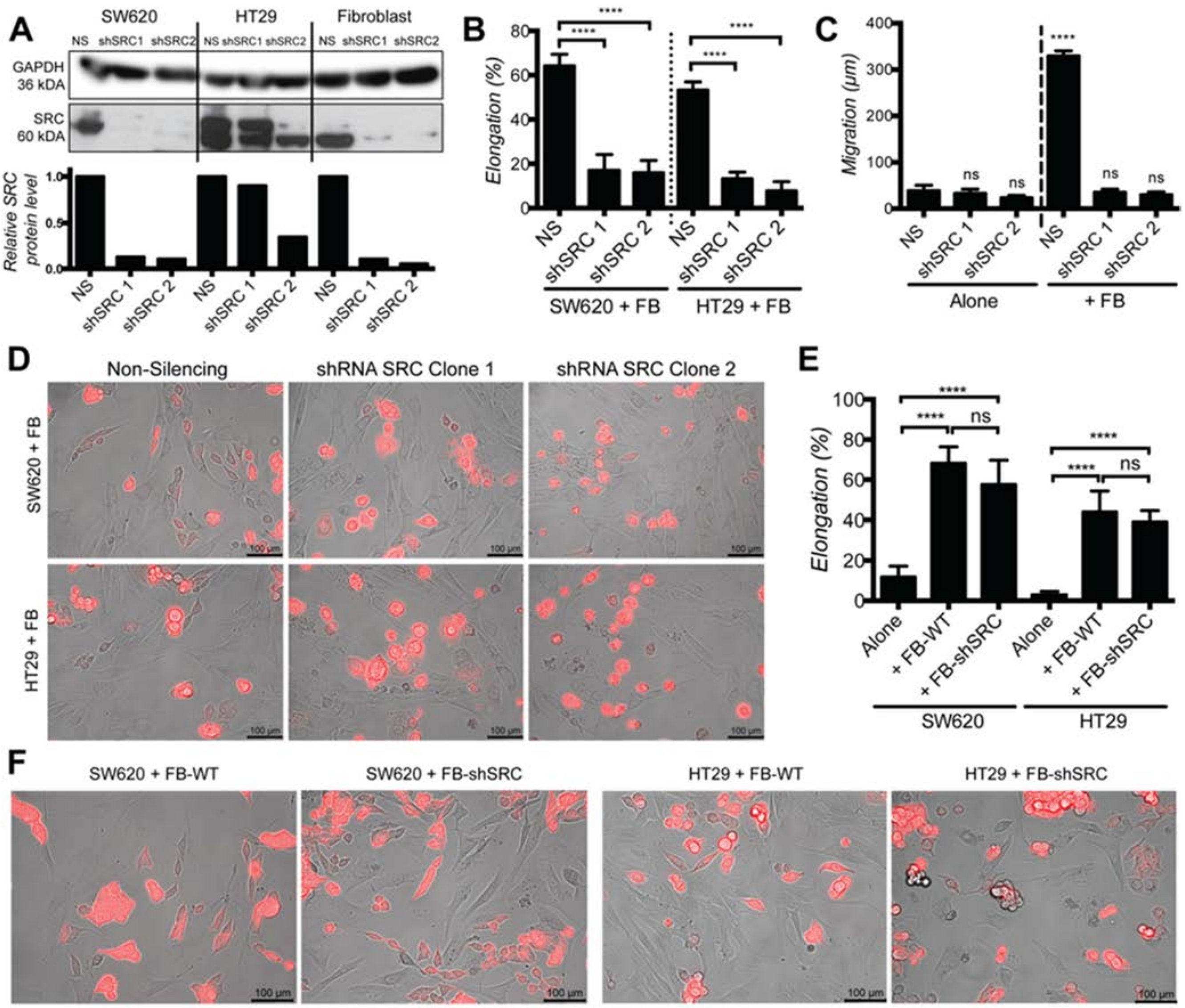

Supplementary Figure S7: SRC knockdown using shRNA constructs demonstrates that effects on cancer cells during co-culture are SRC dependent. A. Western Blot validation of the SRC knockdown for two different clones shRNA for SRC (shSRC 1 and shSRC 2). **B.** Elongation quantification of cancer cells expressing SRC shRNA in presence of fibroblasts (FB). **C.** Motility quantification of SW620 expressing SRC shRNA during 48 hours. **D.** Representatives images of SW620 and HT29 cancer cells expressing LifeAct-mCherry in co-culture with fibroblasts, expressing a non-silencing control (NS) and shRNA for SRC. **E.** Elongation quantification of cancer cells in presence of fibroblasts expressing SRC shRNA. **F.** Representative images of cancer cells expressing LifeAct-mCherry in presence of fibroblasts expressing SRC shRNA. All data are represented as mean +/− SD.

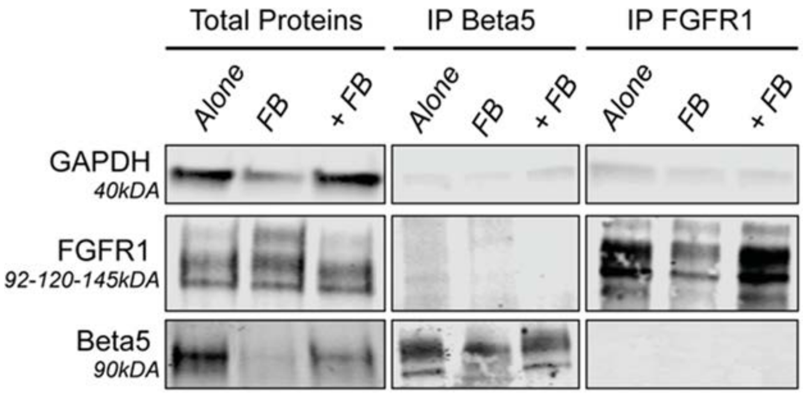

Supplementary Figure S8: Lack of evidence for a direct association between FGFR1 and β_5 integrin by co-immunoprecipitation. Western blotting analysis of FGFR1 and β_5 integrin co-immunoprecipitation in SW620 cultured in presence or absence of fibroblasts and fibroblasts alone under normal condition, of immunoprecipitated β_5 (IP Beta5) and FGFR1 immunoprecipitated (IP FGFR1) material. Western blotting conditions are given on the side.

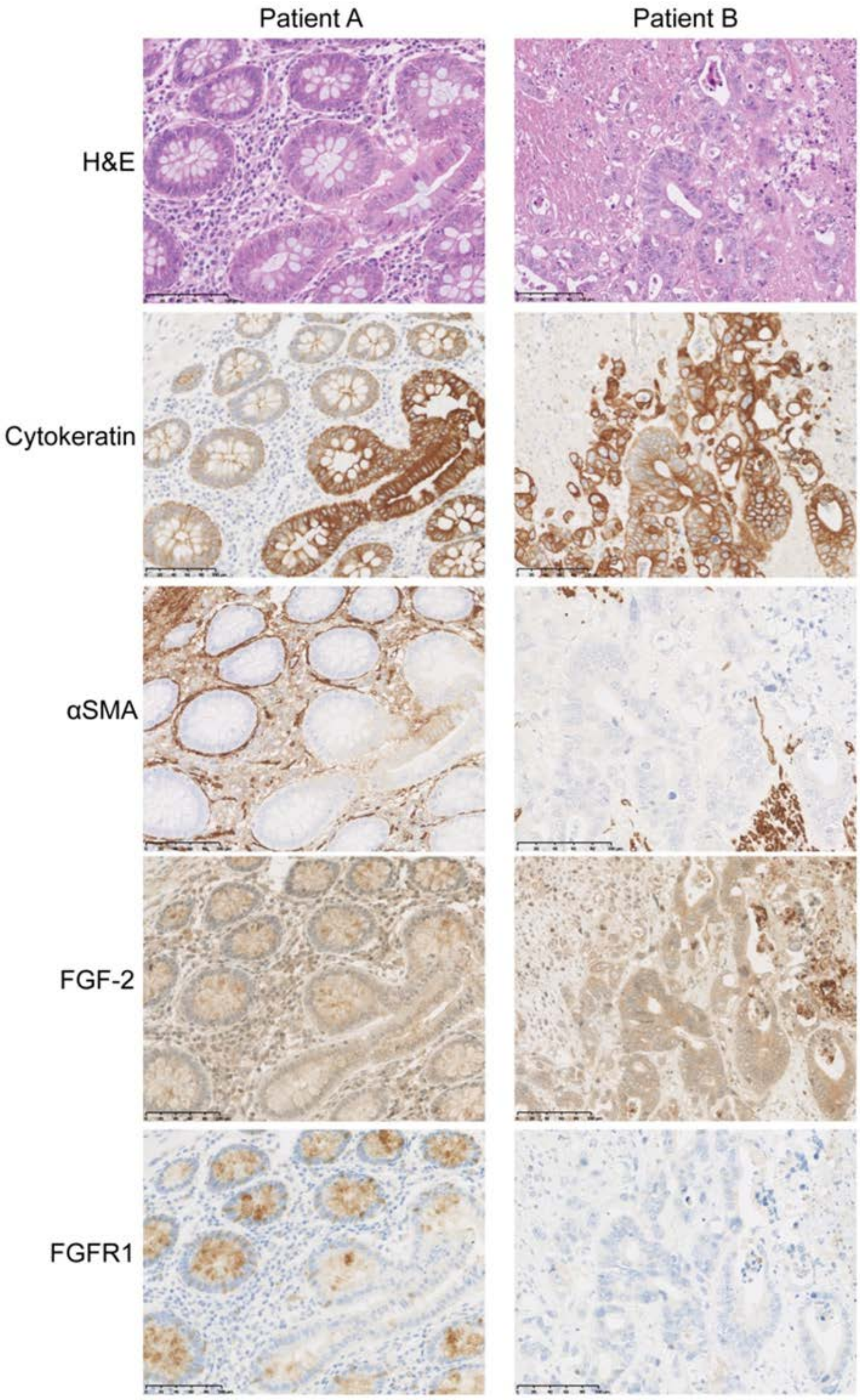

Supplementary Figure S9: Representatives images of FGF-2 and FGFR1 expression in human colon cancer. Representatives images of consecutive sections of non-invasive (Patient A) and invasive (Patient B) lesion of human colorectal cancer stained for the indicated markers.
